# Nonrandom network connectivity comes in pairs

**DOI:** 10.1162/NETN_a_00004

**Published:** 2017-02-01

**Authors:** Felix Z. Hoffmann, Jochen Triesch

**Affiliations:** ^1^Frankfurt Institute for Advanced Studies (FIAS), Johann Wolfgang Goethe University, Frankfurt am Main, Germany; ^2^International Max Planck Research School for Neural Circuits, Max Planck Institute for Brain Research, Frankfurt am Main, Germany

**Keywords:** Nonrandom connectivity, Cortical circuit, Bidirectional connections, Random graph model

## Abstract

Overrepresentation of bidirectional connections in local cortical networks has been repeatedly reported and is a focus of the ongoing discussion of nonrandom connectivity. Here we show in a brief mathematical analysis that in a network in which connection probabilities are symmetric in pairs, *P*_*ij*_ = *P*_*ji*_, the occurrences of bidirectional connections and nonrandom structures are inherently linked; an overabundance of reciprocally connected pairs emerges necessarily when some pairs of neurons are more likely to be connected than others. Our numerical results imply that such overrepresentation can also be sustained when connection probabilities are only approximately symmetric.

## AUTHOR SUMMARY

Understanding the specific connectivity of neural circuits is an important challenge of modern neuroscience. In this study we address an important feature of neural connectivity, the abundance of bidirectionally connected neuron pairs, which far exceeds what would be expected in a random network. Our theoretical analysis reveals a simple condition under which such an overrepresentation of bidirectionally connected pairs necessarily occurs: Any network in which both directions of connection are equally likely to exist in any given pair of neurons, but in which some pairs are more likely to be connected than others, must exhibit an abundance of reciprocal connections. This insight should guide the analysis and interpretation of future connectomics datasets.

## Introduction

Increasing evidence is showing the highly structured nature of cortical microcircuitry (Perin, Berger, & Markram, 2011; Song, Sjöström, Reigl, Nelson, & Chklovskii, 2005). Not every connection is equally likely to be established, but some pairs of neurons are more likely to connect than others. In this context, the relative occurrence of bidirectionally connected pairs has been of particular interest. Using data obtained from paired whole-cell recordings in cortical slices, the amount of bidirectionally connected pairs was compared with the number of reciprocal pairs that one would expect in a random network with the same overall connection probability. The connectivity of Layer 5 pyramidal neurons in the rat visual cortex (Song et al., 2005) and somatosensory cortex (Markram, Lübke, Frotscher, Roth, & Sakmann, 1997; Perin et al., 2011) was shown to have significantly more reciprocal connections than expected.

The prevalence of bidirectional connectivity has since been established as an important indicator of the nonrandomness of a network (Lefort, Tomm, Floyd Sarria, & Petersen, 2009; Bourjaily & Miller, 2011). However, the exact relationship between nonrandomness and relative reciprocity has not been explained. Here, we model cortical circuitry as random networks in which each possible connection has a separate probability to exist. Using this model, we are able to show that any nonrandom connectivity, expressed as higher connection probabilities in some edges and lower probabilities in others, necessarily induces a relative overrepresentation of bidirectional connections, as long as the connection probabilities remain symmetric within pairs. Quantitatively, we analyze reciprocity in networks with one discrete and one continuous distribution of connection probabilities to demonstrate that the relative abundance of bidirectional connections reported in experimental studies can easily be obtained from these models.

### Results

The emergence of nonrandom connectivity patterns can be modeled by assigning each possible connection in a random graph a separate probability to exist. In such a model some connections are more likely to be realized than others, allowing for the encoding of patterns within the specific probabilities of each connection. In the limiting case, each connection either exists or is absent with certainty, representing a blueprint for the network architecture.

To analyze the effect of nonrandom structures within a network—specifically, on the statistics of bidirectionally connected pairs found in the network—we consider a random graph model of *N* neurons in which the probability of node *i* to connect to node *j* is modeled by a random variable *P*_*ij*_. For this analysis, we assume *P*_*ij*_, for *i*, *j* = 1, … , *N* with *i* ≠ *j*, to be identically distributed random variables in [0,1], yielding a probability of connection for each ordered pair of nodes in the graph. Outside of pairs, the random variables *P*_*ij*_ are assumed to be independent—that is, nonequal *P*_*ij*_ and *P*_*kl*_ are independent as long as *i* ≠ *l* or *j* ≠ *k*. Finally, we explicitly exclude self-connections in this model and assume at all times that *i* ≠ *j*.

Given the distributions of connection probabilities, what is then the probability in this model for a randomly selected node to have a projection to another randomly selected node? Since the random variables *P*_*ij*_ are identically distributed, we compute this overall connection probability *μ* easily as the expected value of *P*_*ij*_,

$$\mu =E\left(P_{ij}\right)\text{.}$$ (1)

For example, if the values of *P*_*ij*_ have a probability density function *f* with support in [0,1], we can compute the connection fraction as

$$\mu =\int_{0}^{1}xf\left(x\right)dx\text{.}$$ (2)

In this work, we are interested in the probability *P*_bidir_ that a bidirectional connection exists in a random pair of neurons. We determine *P*_bidir_ as the expected value of the product of *P*_*ij*_ and *P*_*ji*_,

$$P_{\mathrm{bidir}}=E\left(P_{ij}P_{ji}\right)\text{.}$$ (3)

The relative occurrence ϱ of such reciprocally connected pairs compares *P*_bidir_ with the occurrence of bidirectional pairs in an Erdős–Rényi graph, in which each unidirectional connection is equally likely to occur with probability *μ* (Erdős & Rényi, 1959; Gilbert, 1959). The probability that a particular bidirectional connection exists in such a random graph is simply *μ*^2^, and we obtain the relative occurrence as the quotient

$$\varrho =\frac{P_{\mathrm{bidir}}}{\mu^{2}}=\frac{E\left(P_{ij}P_{ji}\right)}{E{\left(P_{ij}\right)}^{2}}\text{.}$$ (4)

Experimental studies in local cortical circuits of rodents have repeatedly reported a rela- tive occurrence of bidirectional connections ϱ > 1 (Markram et al., 1997; Perin et al., 2011; Song et al., 2005). To understand in which cases such an overrepresentation occurs, we consider two cases. In the first case, assume that connection probabilities are independently determined within pairs, as well, meaning that the random variables *P*_*ij*_ and *P*_*ji*_ are independent. Then, because *P*_*ij*_ and *P*_*ji*_ are identically distributed,

$$E\left(P_{ij}P_{ji}\right)=E\left(P_{ij}\right)E\left(P_{ji}\right)=E{\left(P_{ij}\right)}^{2}\text{,}$$ (5)

and we would expect to observe no overrepresentation of reciprocal connections, ϱ = 1. In the second case, assume that connection probabilities are symmetric with in pairs, *P*_*ij*_ = *P*_*ji*_. In this case,

$$P_{\mathrm{bidir}}=E\left(P_{ij}^{2}\right)\text{,}$$ (6)

and the expected relative occurrence of reciprocal connections becomes

$$\varrho =\frac{E\left(P_{ij}^{2}\right)}{E{\left(P_{ij}\right)}^{2}}\text{.}$$ (7)

We note that now any distribution of *P*_*ij*_ with a nonvanishing variance will lead to a relative occurrence that deviates from the Erdős–Rényi graph, because

$$Var\left(P_{ij}\right)=E\left(p_{ij}^{2}\right)-E{\left(p_{ij}\right)}^{2}\text{.}$$ (8)

Moreover, since *x* ↦ *x*^2^ is a strictly convex function, Jensen’s inequality (Cover & Thomas, 2006; Jensen, 1906) yields

$$E\left(P_{ij}^{2}\right)\geq E{\left(P_{ij}\right)}^{2}\text{,}$$ (9)

and we find that ϱ ≥ 1 in networks with symmetric connection probabilities. Jensen’s inequality further states that the equality in (9), and thus ϱ = 1, holds if and only if *P*_*ij*_ follows a *degenerate distribution*—that is, if all *P*_*ij*_ take the identical value *μ*. In the other case, where *P*_*ij*_ takes on more than one value with nonzero probability, we speak of a *nondegenerate distribution*.

As a central result of this study, we thus find that any nondegenerate distribution of symmetric connection probabilities (*P*_*ij*_ = *P*_*ji*_) necessarily induces an overrepresentation of bidirectional connections in the network, ϱ > 1. In other words, in a network in which both directions of connection are equally likely within any given pair, but in which some pairs are more likely to be connected than others, the count of expected reciprocally connected pairs is strictly underestimated by the statistics of an Erdős–Rényi graph with the same overall connection probability E(*P*_*ij*_) = *μ*.

## Upper Bound for ϱ

The overrepresentation of bidirectional connections ϱ in a network is maximal when every connected pair is already a reciprocally connected pair. In terms of the model defined above, this is the case when

$$E\left(p_{ij}p_{ji}\right)=E\left(p_{ij}\right)\text{.}$$ (10)

The relative occurrence of reciprocal connections from (4) then becomes

$$\varrho =\frac{1}{E\left(P_{ij}\right)}=\frac{1}{\mu }\text{.}$$ (11)

Thus, for local cortical circuits of Layer 5 pyramidal neurons with a typical connection probability of *μ* = 0.1 (Song et al., 2005; Thomson, West, Wang, & Bannister, 2002), the network model yields a maximal overrepresentation of ϱ = 10. While this theoretical maximum is unlikely to exist in actual cortical networks, the precise degree of overrepresentation will depend on the specific distribution of connection probabilities in the network. In the following s, we study two generic examples.

## Two-Point Distribution

**Figure 1.**
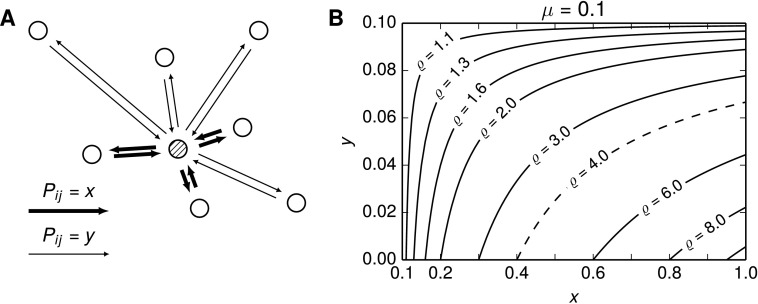
Relative overrepresentation ϱ of bidirectional connections in networks with a fraction of pairs connected with a high probability *x* and the rest of the pairs connected with a low probability *y*. (A) Diagram illustrating the targets with a high chance *x* of connecting (thick arrows) and targets with a low probability *y* of connecting (thin arrows) for a single source node (hatched). (B) Different pairings of *x* and *y* can induce a high relative overrepresentation ϱ in a network with two-point-distributed connection probabilities, $P_{ij}∼T(\frac{\mu -y}{x-y},x,y)$, and a fixed overall connection probability *μ* = 0.1. The dashed line marks an overrepresentation of bidirectional connections of ϱ = 4, observed for Layer 5 pyramidal neurons in rat visual cortex (Song et al., 2005).

The simplest nondegenerate distribution of connection probabilities is a distribution that takes two values *x*, *y*, with probabilities *p* and 1 − *p*, respectively, as illustrated in Figure 1A. This distribution may be seen as a crude approximation to the connection probabilities recently observed in visual cortex as a function of the neurons’ absolute difference in orientation preference, where a “high” connection probability was reported for a difference between 0° and 45° and a “low” probability was seen for cells with a difference of 45°–90° in orientation tuning (Lee et al., 2016).

Formally, let *x*,*y* ∈ [0,1] with *x* > *y* and 0 < *p* < 1. A random variable *X* follows the two-point distribution T(p,x,y) if *P*(*X* = *x*) = *p* and *P*(*X* = *y*) = 1 − *p*.

In our network model, let then the *P*_*ij*_ be T(p,x,y) distributed. The overall connection probability *μ* is

$$\mu =E\left(P_{ij}\right)=px+\left(1-p\right)y\text{.}$$ (12)

Assume again that *P*_*ij*_ = *P*_*ji*_. The relative occurrence of bidirectional connections is given by

$$\varrho =\frac{E\left(P_{ij}^{2}\right)}{\mu^{2}}=\frac{px^{2}+\left(1-p\right)y}{\mu^{2}}\text{.}$$ (13)

Solving (12) for *p* as

$$p=\frac{\mu -y}{x-y}$$ (14)

and inserting this result into (13) yields an expression for the relative overrepresentation that depends on *x*, *y*, and *μ* (see Hoffmann & Triesch, 2016c, SI1),

$$\varrho =\frac{x+y}{\mu }-\frac{xy}{\mu^{2}}\text{.}$$ (15)

Here we fix *μ* = 0.1 in accordance with the overall connection probability found in local circuits of pyramidal cells in the rat visual cortex (Song et al., 2005) and obtain the relative occurrence dependent on the two connection probability values *x* and *y*. Given *x* ≥ *μ*, it follows that *y* ≤ *μ* (see Hoffmann & Triesch, 2016c, SI2) and that the possible values for *x* and *y* are 0.1 ≤ *x* ≤ 1 and 0 ≤ *y* ≤ 0.1. Figure 1B shows contours of ϱ for these (*x*,*y*) pairings, illustrating how different values for the relative overrepresentation of reciprocal connections can be induced by two-point-distributed connection probabilities. We find that in such networks, higher values of ϱ are easily obtained with reasonable network configurations. For example, a relative overrepresentation of ϱ = 4 could be achieved by a two-point distribution of connection probabilities in which one group of neuron pairs is highly connected with probability *x* = 0.7, while the other group of neuron pairs is sparsely connected with probability *y* = 0.05. Collectively, the highly connected pairs then make up less than 8% of all neuron pairs, showing that it is sufficient to have a small subgroup of highly connected neuron pairs to induce a high overrepresentation of bidirectionally connected pairs in the network. For more densely connected networks, *μ* > 0.1, the effect that two distinct connection probabilities have on the overrepresentation of reciprocal connections is reduced (cf. Figure S1), as one would intuitively expect from the dependence of the maximal overrepresentation on *μ* in (11).

## Gamma Distribution

Next, we analyze the relative overrepresentation of bidirectional connections in a network with continuously distributed connection probabilities. The gamma distribution Γ(*α*, *β*) with probability density function

$$f_{\alpha ,\beta }\left(x\right)=\left\{\begin{array}{ll} \frac{1}{\beta^{\alpha }\Gamma \left(\alpha \right)}x^{\alpha -1}e^{-x/\beta }, & x\geq 0,\\ 0, & \mathrm{otherwise}\text{,} \end{array}\right.$$ (16)

allows for the variance Var(*X*) = *αβ*^2^ of a gamma-distributed random variable *X* ∼ Γ(*α*, *β*), to take on different values, while keeping its mean E(*X*) = *αβ* constant (Hogg & Craig, 1978). The exponential distribution emerges as a special case of the gamma distribution (*α* = 1).

To ensure that the randomly drawn connection probabilities lie within the interval [0,1], we here consider a modification to the traditional gamma distribution, in the form of a truncated version. Let *α*, *β* > 0. A random variable *X* follows the truncated gamma distribution Γ^T^(*α*, *β*) if it has the probability density function

$$f_{\alpha ,\beta }^{T}\left(x\right)=\left\{\begin{array}{ll} K_{\alpha ,\beta }\frac{1}{\beta^{\alpha }\Gamma \left(\alpha \right)}x^{\alpha -1}e^{-x/\beta }, & 0\leq x\leq 1,\\ 0, & \mathrm{otherwise}\text{.} \end{array}\right.$$ (17)

The factor *K*_*α*, *β*_ is the inverse of the cumulative probability that *x* ≤ 1 in the untruncated gamma distribution,

$$K_{\alpha ,\beta }={\left(\int_{0}^{1}f_{\alpha ,\beta }\left(x\right)dx\right)}^{-1}\text{,}$$ (18)

and is needed to ensure that

$$\int f_{\alpha ,\beta }^{T}\left(x\right)dx=1\text{.}$$ (19)

Consider the above network model in which the connection probabilities $P_{ij}^{\text{T}}$ are Γ^T^(*α*, *β*) distributed and $P_{ij}^{\text{T}}=P_{ji}^{\text{T}}$. We compute the relative overrepresentation ϱ numerically from

$$\mu =E\left(P_{ij}^{T}\right)=\int_{0}^{1}xf_{\alpha ,\beta }^{T}\left(x\right)dx\text{,}$$ (20)

$$E\left(P_{ij}^{T^{2}}\right)=\int_{0}^{1}x^{2}f_{\alpha ,\beta }^{T}\left(x\right)dx\text{.}$$ (21)

**Figure 2.**
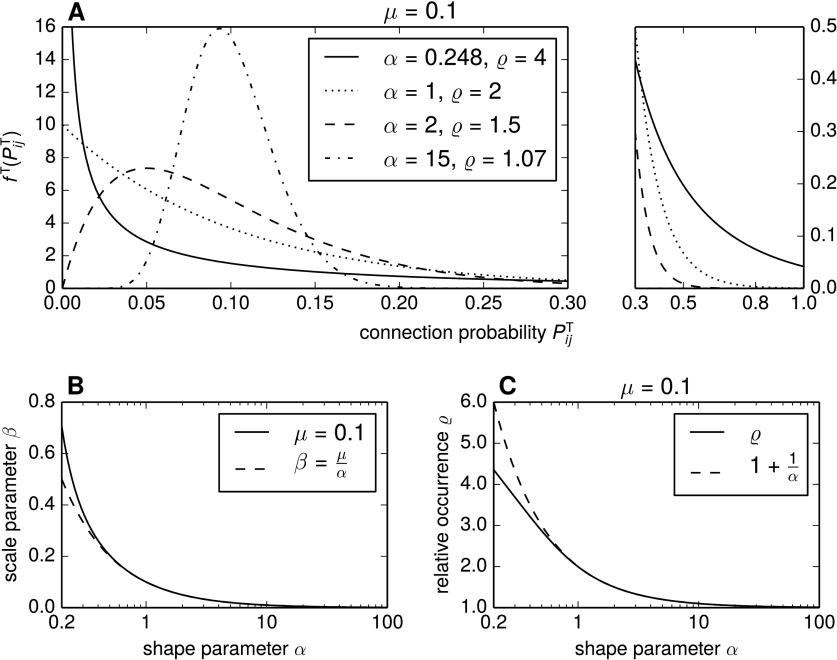
Relative occurrences of bidirectional connections ϱ in networks with gamma-distributed connection probabilities. (A) Probability density functions of the truncated gamma distribution Γ^T^(*α*, *β*) for different shape parameters *α* and the induced relative overrepresentation ϱ in a network with such distributed connection probabilities *P*_*ij*_. For a given *α*, the scale parameter *β* was chosen such that *μ* = 0.1. The plot to the right continues the density functions at a different scale. (B) Contours of *α*, *β* pairings that yield an overall connection probability of *μ* = 0.1. The dashed line shows the approximation $\beta =\frac{\mu }{\alpha }$, where *μ* = 0.1. (C) Relative occurrences ϱ as a function of *α* for fixed *μ* = 0.1. For *α* ≥ 1, this relationship is well approximated by $\varrho \approx 1+\frac{1}{\alpha }$.

Pairings of the shape parameter *α* and the scale parameter *β* are chosen such that the overall connection probability reflects connectivity statistics in local cortical networks, *μ* = 0.1 (Song et al., 2005; Thomson et al., 2002). The probability density functions and the resulting relative overrepresentation of reciprocal connections ϱ for four representative *α*, *β* pairs are shown in Figure 2A. Here, *β* is determined so as to yield *μ* = 0.1 for the given *α*, following the relationship shown in Figure 2B (solid curve).

In the sparse networks we modeled, the tail of the gamma distribution is near zero at $P_{ij}^{\text{T}}=1$ (see Figure 2A). Thus *K*_*α*, *β*_ ≈ 1, and the truncated gamma distribution can be well approximated by the untruncated version. Assuming the connection probabilities to be standard gamma distributed, *P*_*ij*_ ∼ Γ(*α*, *β*), we have

$$E\left(P_{ij}^{2}\right)=Var\left(P_{ij}\right)+E{\left(P_{ij}\right)}^{2}=\alpha \beta^{2}+\alpha^{2}\beta^{2}\text{,}$$ (22)

and thus

$$\varrho =\frac{E\left(P_{ij}^{T^{2}}\right)}{E{\left(P_{ij}^{T}\right)}^{2}}\approx \frac{E\left({P_{ij}}^{2}\right)}{E{\left(P_{ij}\right)}^{2}}=\frac{\alpha^{2}\beta^{2}}{\alpha^{2}\beta^{2}}+\frac{\alpha \beta^{2}}{\alpha^{2}\beta^{2}}=1+\frac{1}{\alpha }=:\tilde{\varrho }\text{.}$$ (23)

The approximation $\varrho \approx \tilde{\varrho }=1+\frac{1}{\alpha }$ works well for *α* ≥ 1, as is shown in Figure 2C.

To induce a high overrepresentation of reciprocal pairs in the network, the gamma distribution of connection probabilities takes a highly skewed shape. To obtain ϱ = 4, only 57% of pairs are expected to have a higher connection probability than 0.01 (*α* = 0.248, *β* = 0.487). Such a situation, in which a large part of all neuron pairs have a small connection probability while some few pairs have a high chance to be connected, is likely to occur if, for instance, the connection probability strongly depends on the spatial separation of the neurons, as was found in Layer 5 excitatory circuits of the rat somatosensory cortex (Perin et al., 2011). Then only nearby neurons are likely to be connected, while the larger part of more distant neurons has a low probability of connection.

## Symmetry of Connection Probabilities in Neural Circuits

In neural circuits, connection probabilities that are equal within pairs but differ across the network are plausible from both an anatomical and a functional perspective. From the anatomical point of view, the distance dependency of connection probabilities mentioned above is a characteristic of cortical circuits that necessarily leads to symmetric probabilities: The distance from the first neuron’s soma to the second neuron’s soma is the same as the distance from the second to the first, resulting in equal probabilities within a pair of neurons when interneuron distance determines the connection probabilities. Regarding the functional perspective, connection probabilities may also depend on the functional properties of the cells in the network. For example, the probability of connections between orientation-tuned cells in the mouse primary visual cortex depends on their absolute difference in orientation tuning (Ko et al., 2011; Lee et al., 2016). Since the absolute difference in orientation tuning will be the same in both directions, connection probabilities can be expected to be equal within a pair of orientation-tuned cells.

However, even when connection probabilities within pairs do not match exactly, an overrepresentation of reciprocal connections is still likely to be observed when connection probabilities follow a nondegenerate distribution. To see this, consider that connection probabilities *P*_*ij*_ are distributed according to some probability density function $f_{P_{ij}}(x)$. As before, we assume that the values of *P*_*ij*_ are independent outside of pairs. In the following discussion, we also assume that *i* > *j* without loss of generality. The expected probability of a reciprocal connection within a pair can then be expressed as

$$E\left(P_{ij}P_{ji}\right)=\int_{0}^{1}\int_{0}^{1}xy\;f_{P_{ij},P_{ji}}\left(x,y\right)dx\;\mathrm{dy}\text{,}$$ (24)

where $f_{P_{ij},P_{ji}}(x,y)$ is the joint probability density function of *P*_*ij*_ and *P*_*ji*_,

$$f_{P_{ij},P_{ji}}\left(x,y\right)=f_{P_{ji}|P_{ij}}\left(x|y\right)f_{P_{ij}}\left(x\right)\text{.}$$ (25)

In the case that *P*_*ij*_ and *P*_*ji*_ are independent, we have $f_{P_{ji}|P_{ij}}(y|x)=f_{P_{ji}}(y),$ and in the case of *P*_*ij*_ = *P*_*ji*_, $f_{P_{ji}|P_{ij}}(y|x)=\delta (y-x)$. Here we propose a model for the conditional density function that transitions between the two extreme cases by multiplying $f_{P_{ji}}(y)$ by the density function of a normal distribution centered around *x*,

$$f_{P_{ji}|P_{ij}}\left(y|x\right)=\frac{1}{N_{\sigma }\left(x\right)}f_{P_{ji}}\left(y\right)\frac{1}{\sigma \sqrt{2\pi }}e^{\frac{{\left(y-x\right)}^{2}}{2\sigma^{2}}}\text{,}$$ (26)

where the additional factor *N*_*σ*_(*x*)^−1^ makes sure that $f_{P_{ji}|P_{ij}}(y|x)$ integrates to 1,

$$N_{\sigma }\left(x\right)=\int_{0}^{1}f_{P_{ji}}\left(z\right)\frac{1}{\sigma \sqrt{2\pi }}e^{\frac{{\left(z-x\right)}^{2}}{2\sigma^{2}}}dz\text{.}$$ (27)

Indeed, as the standard deviation *σ* of the modulating normal distribution increases, $f_{P_{ji}|P_{ij}}(y|x)$ approaches $f_{P_{ji}}(y)$, and in the limit *σ* → 0 we have

$$\lim_{\sigma \to 0}f_{P_{ji}|P_{ij}}\left(y|x\right)=\delta \left(y-x\right)\text{.}$$ (28)

**Figure 3.**
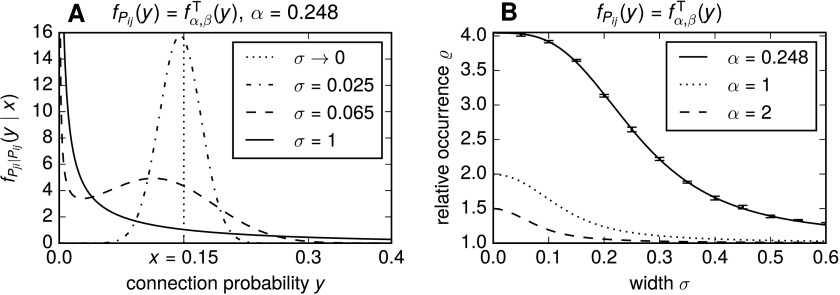
Relative overrepresentation of bidirectional connections ϱ is sustained when connection probabilities are only approximately symmetric in pairs. (A) Illustration that the conditional density function $f_{P_{ji}|P_{ij}}(y\;|\;x)$ in (26) transitions from equality of the random variables *P*_*ij*_ and *P*_*ji*_ to independence with increasing *σ*. We use $f_{P_{ij}}(y)=f_{\alpha ,\beta }^{\text{T}}(y)$, with *α* = 0.248 and *β* such that E(*P*_*ij*_) = 0.1. For the illustration, *P*_*ij*_ was fixed as *x* = 0.15. Already for *σ* = 1, the conditional density function becomes visually indistinguishable from *f*_*α*, *β*_^T^(*y*) (cf. Figure 2A). (B) Relative occurrences of reciprocally connected pairs ϱ as a function of *σ*. The curves for *α* = 1 and *α* = 2 show numerical solutions of (29) with $f_{P_{ij}}(y)=f_{\alpha ,\beta }^{\text{T}}(y)$, where *β* was chosen such that E(*P*_*ij*_) = 0.1. Relative reciprocal pair counts from generated networks following the model matched these theoretical curves (data not shown). For *α* = 0.248, random variables with the respective probability density functions were sampled and the average ϱ was computed via (29) using the sample means. Error bars show *SEM*s; the curve for *α* = 0.248 (solid line) was fitted to the data points and is purely for illustrative purposes.

In Figure 3A, the conditional density functions for various *σ* are shown for the truncated gamma distribution. For low values of *σ*, the conditional density function resembles a narrow Gaussian around *x*, reflecting approximately symmetric connection probabilities. For *σ* > 1, on the other hand, $f_{P_{ji}|P_{ij}}(y|x)$ becomes virtually indistinguishable from *f*_*α*, *β*_^T^(*y*), reflecting the independence of *P*_*ji*_ from *P*_*ij*_.

Finally, we employ the model to examine how the relative overrepresentation of bidirec- tional connections ϱ changes with the degree of symmetry in the connection probabilities within a pair of neurons. To do so, ϱ is computed as a function of *σ* for a given distribution of *P*_*ij*_ using

$$\varrho =\frac{E\left(P_{ij}P_{ji}\right)}{\mu^{2}}\text{,}$$ (29)

the numerator is defined through (24–26), and the overall connection probability *μ* is calculated as

$$\mu =\frac{1}{2}\int_{0}^{1}x\;f_{P_{ij}}\left(x\right)dx+\frac{1}{2}\int_{0}^{1}f_{P_{ij}}\left(x\right)\int_{0}^{1}y\;f_{P_{ji}|P_{ij}}\left(y|x\right)dx\;\mathrm{dy}\text{.}$$ (30)

since half of the connection probabilities are drawn according to $f_{P_{ij}}$ and the other half are drawn according to $f_{P_{ji}|P_{ij}}$ given a particular value of *x* drawn according to $f_{P_{ij}}$. Figure 3B shows the change of ϱ with *σ* for connection probabilities *P*_*ij*_ following a truncated gamma distribution Γ^T^(*α*, *β*). For the three parameter sets chosen, we see that a strong overrepresentation of bidirectional connections is sustained when connection probabilities are only approximately symmetric in pairs. Furthermore, as long as *P*_*ji*_ is at biased to take similar values to *P*_*ij*_, an overrepresentation of ϱ > 1 can be observed, implying that effects such as distance dependency or the dependence on the absolute difference in orientation tuning of connection probabilities will tend to increase the relative occurrence of bidirectional connections, even when other effects are also influencing the neurons’ connection probabilities.

## DISCUSSION

Experimental evidence suggests that any pair of excitatory cells within a cortical column has contact points between axon and dendrite close enough to support a synaptic connection between the cells (Kalisman, Silberberg, & Markram, 2005; Stepanyants, Tamás, & Chklovskii, 2004). Despite this potential “all-to-all” connectivity, only a small fraction of the contacts are realized as functional synapses. Uncovering the principles underlying which contact points get utilized for synaptic transmission is crucial for our understanding of the structure and function of the local cortical circuits in the mammalian brain.

Local networks in the rat visual and somatosensory cortices have been shown to feature nonrandom structure (Perin et al., 2011; Song et al., 2005), and much attention has been given to bidirectionally connected neuron pairs that occur more often than would be expected from random connectivity (Bourjaily & Miller, 2011; Clopath, Büsing, Vasilaki, & Gerstner, 2010; Miner & Triesch, 2016). In this study we have shown a condition under which nonrandom network structure and the occurrence of reciprocally connected pairs are inherently linked; a relative overrepresentation of bidirectional connections arises necessarily in networks with a nondegenerate distribution of symmetric connection probabilities. The absence of an overabundance of reciprocal pairs, on the other hand, as for example is found in the intralayer connectivity of the mouse C2 barrel column (Lefort et al., 2009), points toward either a truly random network or an asymmetry in the connection probabilities.

Quantitatively, a network in which connection probabilities take on one of two values is easily able to account for even the highest values of overrepresentation reported. A network with such a two-point distribution of connection probabilities might occur naturally, where the probability of connection depends on whether a given pair of neurons shares a certain feature—for example, have or do not have similar orientation preferences (Lee et al., 2016).

A continuous distribution in connection probabilities, on the other hand, might occur when pair connectivity depends on a continuous parameter, such as the interneuron distance or the neurons’ ages. We showed that networks in which connection probabilities follow a gamma distribution can also have a high relative occurrence of reciprocally connected pairs; however, in this case a larger fraction of pairs remain unconnected with a very high probability.

It is likely that a combination of such effects determines the connection probabilities in local cortical networks. Importantly, we showed that as long as this probability is symmetric for pairs, any such effect that creates a nondegenerate distribution of probabilities will cause an increase of the reciprocity in the network.

Our results confirm the intuitive notion that reciprocity is favored in symmetric networks, whereas asymmetric probabilities of connection inhibit the occurrence of bidirectionally connected pairs. Network models with symmetric connectivity, such as Hopfield nets, generally excel at memory storage and retrieval through fixed-point attractor dynamics (Hopfield, 1982), while asymmetric network models such as synfire chains are suitable for reliable signal transmission (Abeles, 1982; Diesmann, Gewaltig, & Aertsen, 1999). This suggests the intriguing possibility that one may be able to infer the nature of the computations in a neural circuit on the basis of certain statistics of its connectivity, such as the abundance of bidirectionally connected pairs.

In conclusion, the present study puts the overrepresentation of bidirectional connections found in local cortical circuits in a new light. If connection probabilities are symmetric in pairs, the overrepresentation emerges as a symptom of any form of nonrandom connectivity. It is thus crucial for both future experimental and modeling studies to develop a more refined view of nonrandom network connectivity that goes beyond simple pair statistics. Focusing on higher-order connectivity patterns and taking into account the actual synaptic efficacies seem promising avenues for future research into the nonrandom wiring of brain circuits.

## SUPPLEMENTARY INFORMATION

The supplementary information document for references SI1 and SI2 and for Figure S1 is available online at doi:10.6084/m9.figshare.3501860. Python code for the numerical comp- utations is available as a GitHub repository, archived along with the generated data at doi:10.5281/zenodo.200368. A website documenting the code can be found at https://non-random-connectivity-comes-in-pairs.github.io/

## ACKNOWLEDGMENTS

The authors thank the anonymous reviewers for their helpful and constructive comments on earlier versions of this article.

## AUTHOR CONTRIBUTIONS

J.T. is supported by the Quandt Foundation. F.Z.H. and J.T. conceived and designed the study. F.Z.H. carried out the formal analysis and performed the simulations. And both F.Z.H. and J.T. wrote the manuscript.

## TECHNICAL TERMS

Jensen’s inequality: If *φ* is a convex function and *X* an integrable random variable, then *φ*(E(*X*)) ≤ E(*φ*(X)), where E(*X*) denotes the expected value of *X.*
Cortical microcircuitry: The set of neuron-to-neuron connections in a small subset of cells in the cortex.
Support: The subset of the domain of a real-valued function whose elements are not mapped to zero.
Erdős–Rényi graph: Randomly wired network in which each possible connection has the same probability to exist.
Orientation preference: The property of neurons in primary visual cortex to respond strongly to visual stimuli with a certain orientation appearing inside their receptive fields.
